# A Smartwatch-Based Framework for Real-Time and Online Assessment and Mobility Monitoring

**DOI:** 10.1093/geroni/igaa057.2895

**Published:** 2020-12-16

**Authors:** Yashaswi Karnati, Rohith Reddy Kessi Reddy, Aseem Thakkar, Matthew McConnell, Tonatiuh Mendoza, Mamoun Mardini, Todd Manini, Sanjay Ranka

**Affiliations:** 1 University of Florida, Gainesville, Florida, United States; 2 The University of Florida, Gainesville, Florida, United States

## Abstract

Pervasive computing is changing the monitoring landscape for patients to communicate their healthcare information in real-time to clinicians and researchers. We developed a framework based on a smartwatch application allowing researchers to execute a study that is customized to their needs. The application is configured to collect patient generated data in remote settings from both sensor-based (location and movement) and user-reported health factors through the visual display. For example, data are used to investigate concurrent symptoms and mobility patterns in free-living conditions. To support the collection and analysis of this data in a robust and scalable fashion, we have developed an event driven, serverless computing platform using Amazon cloud services. This system also allows multiple campaigns to run concurrently each under the auspices of a different researcher. The framework is ideal for harnessing and scaling the utilization of smart wearable devices in research and clinical settings.

